# Chronic replication stress invokes mitochondria dysfunction via impaired parkin activity

**DOI:** 10.1038/s41598-024-58656-w

**Published:** 2024-04-03

**Authors:** Tsuyoshi Kawabata, Reiko Sekiya, Shinji Goto, Tao-Sheng Li

**Affiliations:** Department of Stem Cell Biology, Atomic Bomb Disease Institute, Nagasaki University, Nagasaki, Japan

**Keywords:** Autophagy, Organelles, Senescence

## Abstract

Replication stress is a major contributor to tumorigenesis because it provides a source of chromosomal rearrangements via recombination events. *PARK2*, which encodes parkin, a regulator of mitochondrial homeostasis, is located on one of the common fragile sites that are prone to rearrangement by replication stress, indicating that replication stress may potentially impact mitochondrial homeostasis. Here, we show that chronic low-dose replication stress causes a fixed reduction in parkin expression, which is associated with mitochondrial dysfunction, indicated by an increase in mtROS. Consistent with the major role of parkin in mitophagy, reduction in parkin protein expression was associated with a slight decrease in mitophagy and changes in mitochondrial morphology. In contrast, cells expressing ectopic *PARK2* gene does not show mtROS increases and changes in mitochondrial morphology even after exposure to chronic replication stress, suggesting that intrinsic fragility at *PARK2* loci associated with parkin reduction is responsible for mitochondrial dysfunction caused by chronic replication stress. As endogenous replication stress and mitochondrial dysfunction are both involved in multiple pathophysiology, our data support the therapeutic development of recovery of parkin expression in human healthcare.

## Introduction

Cell division requires a copy of the genome that should be strictly regulated; otherwise, any alterations in genomic information can cause deleterious effects on daughter cells. Nonetheless, cells are constitutively exposed to blockades that halt the progression of replication forks^1^. Stalled replication forks are replication intermediate structures that act as substrates for recombination events and cause gross chromosomal rearrangements, missegregation, and instabilities^2^. Specific loci called common fragile sites (CFSs) are severely targeted by replication stress because of their low density of replication origins, termed replication origin deserts^3^. Some tumor suppressor genes are encoded by CFSs. The *PARK2* locus is one of the CFSs and is known to be rearranged by replication stress induced by the replication inhibitor aphidicolin (APH)^3^. As *PARK2* encodes parkin, a major regulator of mitochondrial homeostasis that promotes selective autophagy targeting mitochondria, called mitophagy^4^, any genetic or epigenetic alteration on *PARK2* locus may potentially cause mitochondrial dysfunction. Considering a higher replication stress induced by oncogene expression in precancerous lesions and a loss of *PARK2* expression in many cancer cells, replication stress could lead to mitochondrial dysfunction with higher reactive oxygen species (ROS) production, a source of endogenous mutagenesis and carcinogenesis. However, it has not been well tested whether replication stress contributes to tumorigenesis in the context of mitochondrial alteration. Additionally, it has not been investigated whether replication stress may promote parkin-independent mitophagy which works in the absence of functional parkin to maintain mitochondrial homeostasis. Moreover, the maintenance of mitochondrial integrity is crucial for many biological events and for the suppression of a wide range of diseases, including cancer, Parkinson’s disease, and amyotrophic lateral sclerosis^5^. Therefore, it is important for human healthcare to determine the mechanisms by which endogenous replication stress affects mitochondrial homeostasis.

In this report, we showed that chronic replication stress causes a fixed reduction in parkin protein expression, leading to mitochondrial dysfunction. We observed that mitochondrial ROS (mtROS) was upregulated in cells recovered from constitutive exposure to replication stress, which may contribute to cancer development associated with replication stress.

## Results

### Replication stress causes reduced parkin protein expression, which persists even after prolonged recovery culture

**Figure 1:**
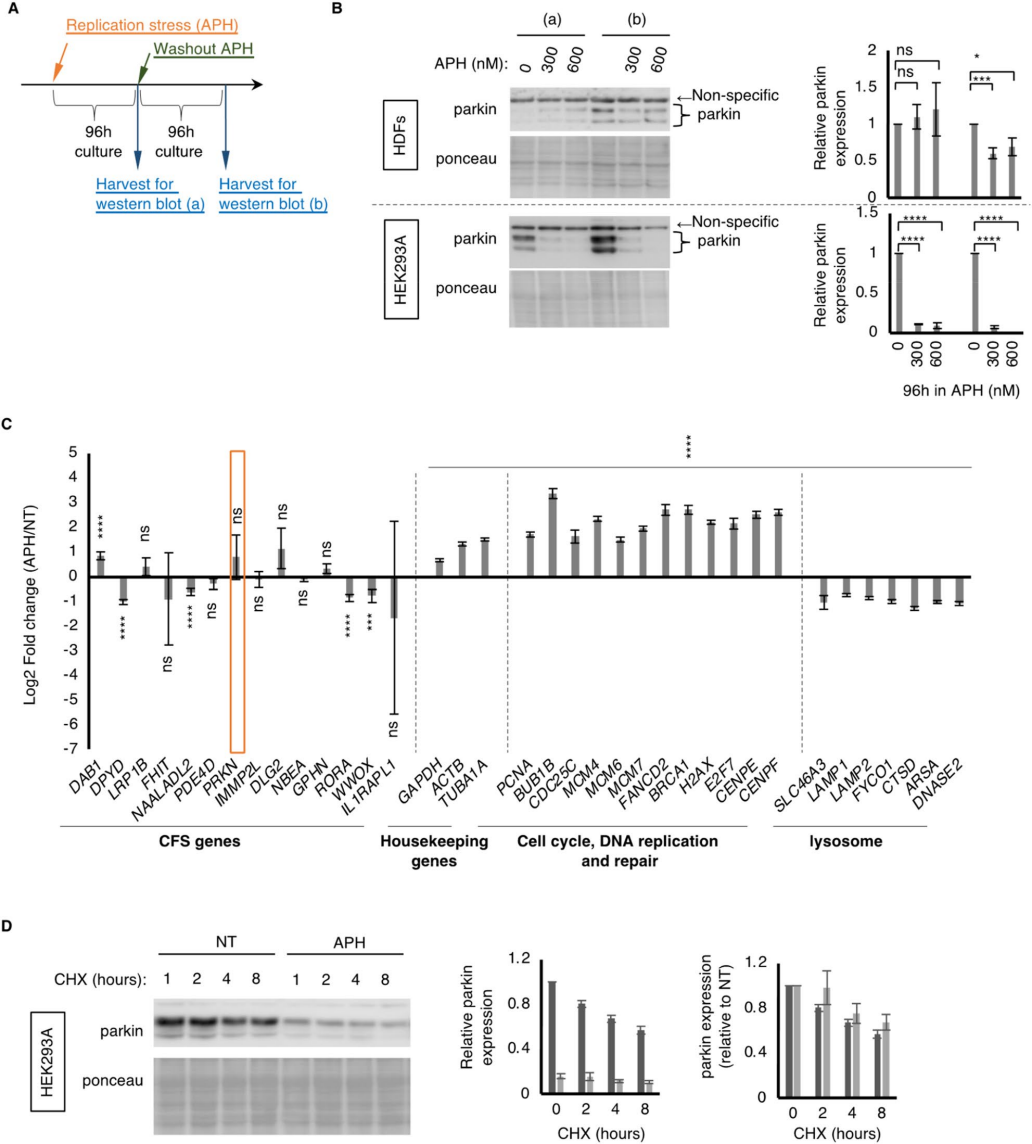
Constitutive replication stress and recovery causes fixed reduction in parkin expression in HDFs. (**A**) Schematics of exposure of HDFs with replication stress by APH treatment. (**B**) Representative image (left) and quantification (right) of immunoblots showing expression level of parkin. Ponceau indicates staining of a membrane before immunostaining used as loading control. Error bar: SEM of at least 3 independent experiments. ns: p > 0.05, *: p < 0.05, ***: p < 0.005, ****: p < 0.001 (One-way ANOVA). (**C**) Summary of RNA-seq experiments showing mRNA expression of genes of each category indicated in the HDF cells exposed to APH, normalized by non-treated controls. *PRKN* (*PARK2*) is highlighted by orange rectangle. Error bars indicates SEM of 3 independent experiments. ns: p > 0.05, ***: p < 0.005, ****: p < 0.001 (One-way ANOVA). (**D**) Representative image (left) and quantification (right) of immunoblots showing expression level of parkin in the HEK293A cells treated with 50 µg/ml of cycloheximide (CHX) for indicated hours. APH indicates 300 nM APH for 96 h followed by recovery culture for 96 h. Error bars indicates SEM of 3 independent experiments.

To understand the effect of replication stress on parkin expression, human dermal fibroblasts were exposed to aphidicolin (APH), an inhibitor of replicative polymerase, and parkin expression was observed by western blotting. We used human dermal fibroblasts immortalized by *hTert*, but not cancer cell lines such as HeLa cells, because cancers are often mutated in and lose expression of *PARK2* gene, possibly due to the fragility of the *PARK2* locus and long-term constitutive exposure of cancer cells to endogenous replication stress that is inherited into subsequent subpopulations^6–11^. We used 300 nM or 600 nM of APH, which does not completely stop the progression of replication forks but causes reduced fork velocity and increased rate of fork stalling^12^. It does not cause cell cycle arrest but induces chromosomal missegregation during the M-phase and chromosomal rearrangements^12–16^. It also causes the expression of fragile sites that may potentially alter the genomic information of *PARK2* loci^17,18^. We tested both acute effects (immediately after 96 h of treatment) and fixed effects (after 96 h of culture recovery). We found that parkin expression was not significantly changed after 96 h of exposure to APH (Figs. 1A, B, S1).

However, after an additional 96 h of recovery culture, the protein levels of parkin were significantly reduced in the cells exposed to APH (Fig. 1B). This is not only specific to HDF, as HEK293A (human embryonic kidney) cells treated with the same treatment showed similar but more severe effects on APH (Fig. 1B), most likely due to the higher proliferative capacity of HEK293A cells than HDFs, which increases the frequency of fork stalling and collapse. This result indicates that chronic replication stress caused a fixed reduction in parkin protein expression. To determine whether the reduced parkin protein stemmed from reduced transcription of *PARK2*, we carried out RNA-seq experiments using HDF exposed to 96 h APH followed by 96 h recovery. HEK293A and HEK293 cells treated with chronic APH showed significantly lower mRNA expression than sham-treated cells (Fig. S1B)^19^, as HEK293A cells showed a greater effect on parkin protein expression than HDF cells. Unexpectedly, we found that parkin mRNA expression in APH-exposed cells was not statistically different from that in untreated controls. This was because of the high variability in parkin mRNA expression after APH treatment (Fig. 1C). Interestingly, many other genes located on other CFSs showed similar variability, with four genes showing significant reduction, one gene showing a significant increase, and nine genes showing no significant change out of 14 genes, while housekeeping genes showed consistent results among the experiments (Fig. 1C, Table S1). To determine whether reduced parkin protein expression might be due to any alteration in protein stability after translation, we performed a cycloheximide chase assay using HEK293A cells treated with cycloheximide for 1, 2, 4, and 8 h. We found that the degradation kinetics of parkin in HEK293A cells treated with chronic APH were comparable to those of untreated cells (Fig. 1D), suggesting that the reduced parkin protein expression caused by chronic replication stress may stem from any issue(s) before translation. We speculate that this inconsistency between mRNA expression and protein expression of parkin in cells treated with chronic APH could be due to unknown issue(s) in quality of mRNA that may stem from fragility of common fragile sites, as this is the locus with a high rate of mutagenesis caused by recurrent recombination and rearrangements that are associated with replication by error-prone polymerase^20–22^. Thus, we supposed that the quality, rather than quantity, of *PARK2* mRNA could be more damaged in this condition, which results in a more significant effect on the readout. To characterize the cells exposed to chronic replication stress, we performed annotation of gene expression profiles using DAVID (https://david.ncifcrf.gov/summary.jsp) and found that APH-treated cells showed constitutive upregulation of genes involved in the cell cycle, DNA replication, and DNA repair, and downregulation of genes involved in lysosomal activity. The former indicates that exposure to chronic replication stress may induce a stress response that protects genomic information from chromosomal rearrangement. Because the lysosomal pathway is regulated by cell cycle regulators such as mTOR and p53^23,24^, these downregulations could be a byproduct of the pro-proliferative alteration of APH-exposed cells. As lysosomes are one of the major degradative pathways that are also involved in mitochondrial homeostasis through mitophagy, this alteration could be taken into account in any changes in mitochondrial function in APH-treated cells.

These results indicate that a fixed change in genomic information at the *PARK2* locus causes chronic exposure to replication stress, resulting in a fixed reduction in Parkin protein expression, which is most likely caused by genomic alteration by replication stress, while other physiological alterations could also be involved in this alteration.

### Chronic exposure of HDF to replication stress causes elevated levels of ROS in mitochondria, whereas acute replication stress does not

**Figure 2:**
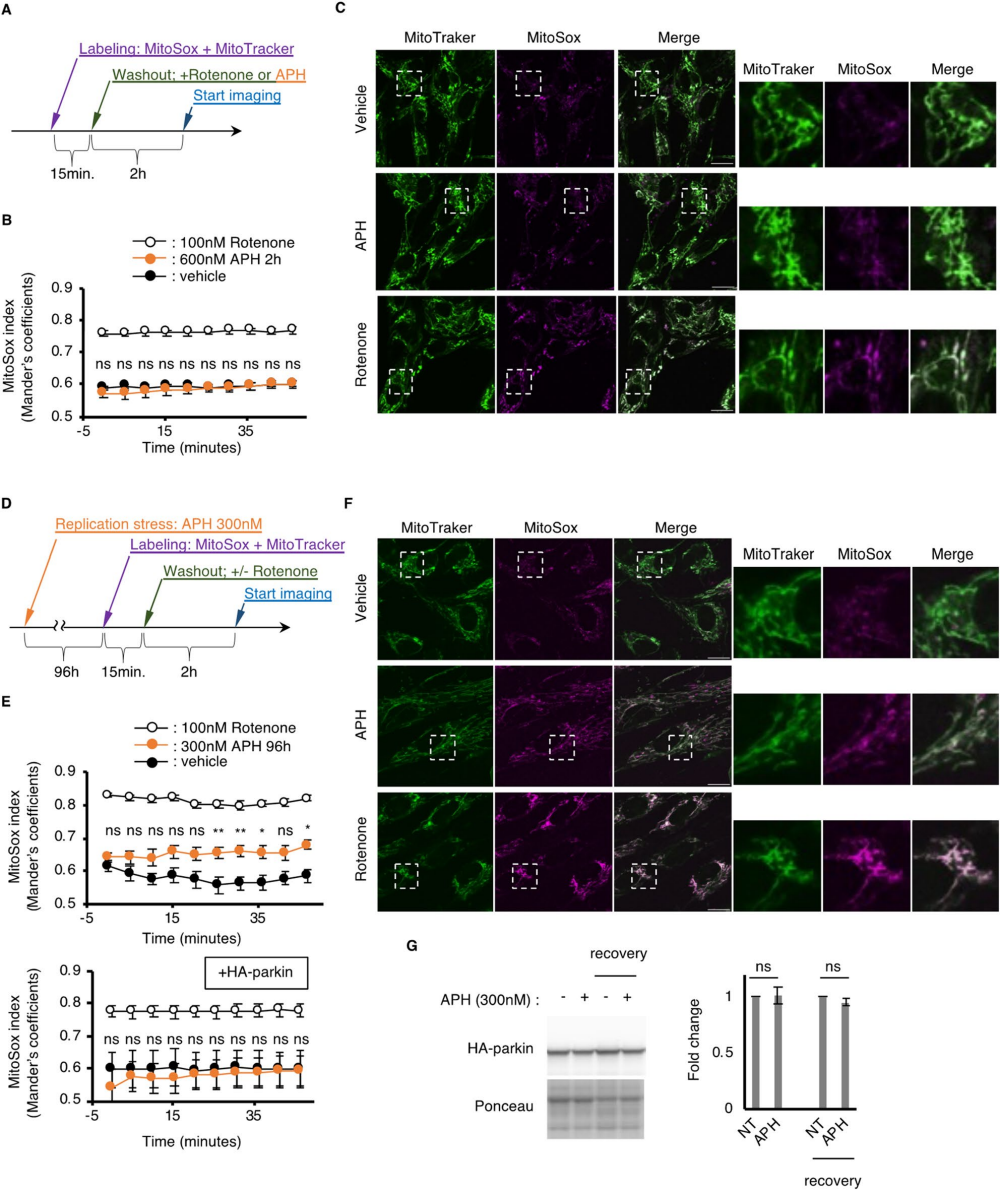
Constitutive replication stress and recovery causes elevated mtROS expression in HDFs. (**A**) Schematics of exposure of HDFs with acute replication stress (APH) or 100 nM of Rotenone, an inhibitor of mitochondrial electron transport chain complex I. (**B**) Sequential progression of mtROS indicated by mitoSOX signal normalized by mitochondrial signal indicated by MitoTracker green. Error bar: SEM of at least 3 independent experiments. ns: p > 0.05, *: p < 0.05, **; p < 0.01, (One-way ANOVA). (**C**) Representative image of microscopic observation of the HDFs labeled with MitoTracker and MitoSOX. Scale bar: 20 µm. (**D**) Schematics of exposure of HDFs with constitutive replication stress (APH). (**E**) Sequential progression of mtROS indicated by mitoSOX signal normalized by mitochondrial signal indicated by MitoTracker green. Error bar: SEM of at least 3 independent experiments. ns: p > 0.05, *: p < 0.05, **; p < 0.01, (One-way ANOVA). (**F**) Representative image of microscopic observation of the HDFs labeled with MitoTracker and MitoSOX. Scale bar: 20 µm. (**G**) Representative image (left) and quantification (right) of immunoblots showing expression level of HA-parkin. Ponceau indicates staining of a membrane before immunostaining used as loading control. Error bar: SEM of at least 3 independent experiments. ns: p > 0.05, student’s t-test.

Next, we determined the effect of parkin reduction by replication stress on mitochondrial homeostasis. We measured the mitochondrial ROS (mtROS) levels, which are elevated by mitochondrial dysfunction. We labeled mitochondria in live HDFs under culture conditions using a confocal microscope system simultaneously with mitoSOX, an mtROS indicator, and MitoTracker, a mitochondrial indicator that was used as an internal control for mitoSOX. First, we confirmed that acute replication stress does not cause significant changes in mtROS expression (Fig. 2A–C). However, mtROS is significantly increased after 96 h of recovery culture (Fig. 2D–F). Since parkin is a major caretaker of mitochondrial homeostasis^25^, we expected that a slight but significant reduction in parkin by replication stress may be responsible for this increase in mtROS. Thus, we introduced an ectopic expression construct of HA-tagged parkin in HDFs and tested the causality of parkin reduction. We confirmed that the expression of ectopic HA-parkin was not reduced even after replication stress, in contrast to endogenous parkin expression, possibly because of the fragility of the genomic locus of endogenous *PARK2* (Figs. 2G, S2). We found that HDF with HA-parkin did not cause any increase in mtROS after replication stress even after 96 h of recovery culture (Fig. 2E). These data suggest that replication stress causes mitochondrial dysfunction that stems from aberrant parkin expression.

### Chronic exposure of HDF to replication stress does not cause major defects in mitophagy or DRP1 phosphorylation

**Figure 3:**
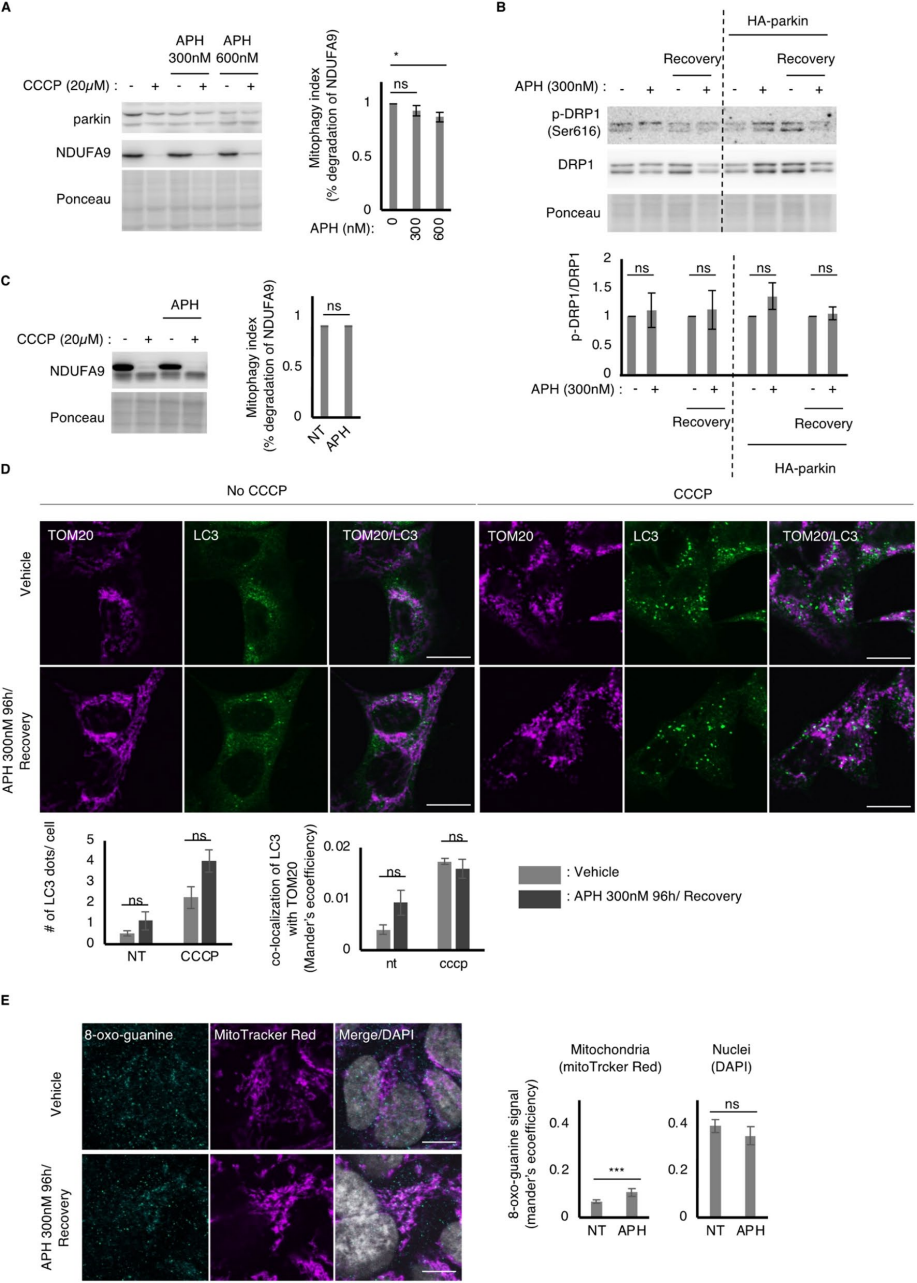
Constitutive replication stress and recovery causes fixed reduction in parkin expression in HDFs. (**A**) Representative image (left) of immunoblots showing expression level of parkin and NDUFA9 in the HDF cells. HDFs were treated with indicated dose of APH for 96 h, followed by recovery culture without APH for 96 h and harvest for western blot. Ponceau indicates staining of a membrane before immunostaining used as loading control. Right bar graph shows mitophagy index indicated by % degradation of NDUFA9 in the cells with indicated dose of CCCP for 24 h. Error bar: SEM of at least 3 independent experiments. ns: p > 0.05. *: p < 0.05, Student’s t-test. (**B**) Representative image (left) and quantification (right) of immunoblots showing DRP1 expression or phosphorylation level of DRP1. HDFs were treated with indicated dose of APH for 96 h, followed by recovery culture without APH for 96 h and harvest for western blot. Ponceau indicates staining of a membrane before immunostaining used as loading control. Error bar: SEM of at least 3 independent experiments. *: p > 0.05, Student’s t-test. (**C**) Representative image (left) of immunoblots showing expression level of parkin and NDUFA9 in HEK293A cells. HEK293A cells were treated with indicated dose of APH for 96 h, followed by recovery culture without APH for 96 h and harvest for western blot. Error bar: SEM of at least 3 independent experiments. ns: p > 0.05. Student’s t-test. (**D**) Representative images (top) and quantifications (bottom) of LC3 (autophagosome) and TOM20 (mitochondria) localizations in the HEK293A cells treated with 20 µM CCCP for 6 h. The # of LC3 dots and co-localization of LC3 with TOM20 were analyzed. Error bar: SEM of at least 3 independent experiments. ns: no significance, Student’s t-test. Scale bar: 20 µm. (**E**) Representative images (left) and quantifications (right) of 8-oxoguanine and MitoTracker Red in the HEK293A cells. Both 8-oxoguanine signals on MitoTracker Red and DAPI (nuclei) were analyzed. ns: p > 0.05, ***: p < 0.005, Student’s t-test. Scale bar: 20 µm.

Next, we sought to determine the mechanism by which replication stress-induced reduction in parkin expression causes mitochondrial dysfunction. Parkin regulates mitophagy, a major pathway for maintaining mitochondrial homeostasis^25^. We measured mitophagy levels in HDF exposed to chronic replication stress and observed a slight reduction in mitophagy activity (Figs. 3A, S3). However, overall mitochondrial degradation was not severely compromised in APH-treated HDFs, indicating that residual parkin expression is sufficient to carry out a large portion of stress-induced mitophagy. As mitochondrial fission promotes mitophagy by generating daughter mitochondria with low membrane potential that can be targeted and sequestered by autophagosome, we measured phosphorylation of DRP1, a pivotal protein for mitochondrial fission^26^. We observed no significant changes in the phosphorylation of DRP1 (Figs. 3B, S4), indicating that DRP1-mediated pathway is intact in the APH-exposed HDFs. As the degree of parkin protein reduction upon chronic APH treatment was greater in HEK293A cells than in HDF, we determined whether chronic APH treatment may cause any changes in mitophagy activity in HEK293A cells. As was the case with HDF cells, we found that the degradation of mitochondrial protein upon CCCP treatment was not significantly changed in APH-treated cells (Fig. 3C). Furthermore, immunofluorescence staining showed that LC3 dot formation was induced by CCCP in APH-treated HEK293A cells at a level comparable to that in non-treated cells, suggesting that autophagosome formation in response to mitochondrial damage was intact in APH-treated HEK293A cells (Fig. 3D), although parkin expression was largely impaired. Consistently, LC3 localization in mitochondria in response to CCCP was not impaired by chronic APH treatment (Fig. 3D). Even though healthy mitophagy activity remains in cells exposed to chronic replication stress, we found that 8-oxoguanine accumulation in mitochondria, but not in nuclei, is increased in HEK293A cells exposed to chronic replication stress (Fig. 3E). These data suggest that the reduction in Parkin protein levels caused by chronic replication stress does not completely disrupt mitophagy activity at least under these experimental conditions, but damages mitochondrial homeostasis. This is not surprising because parkin maintains mitochondrial homeostasis not only via mitophagy, but also by regulating multiple pathways, including the regulation of mitochondrial fission and fusion^27–29^.

**Figure 4:**
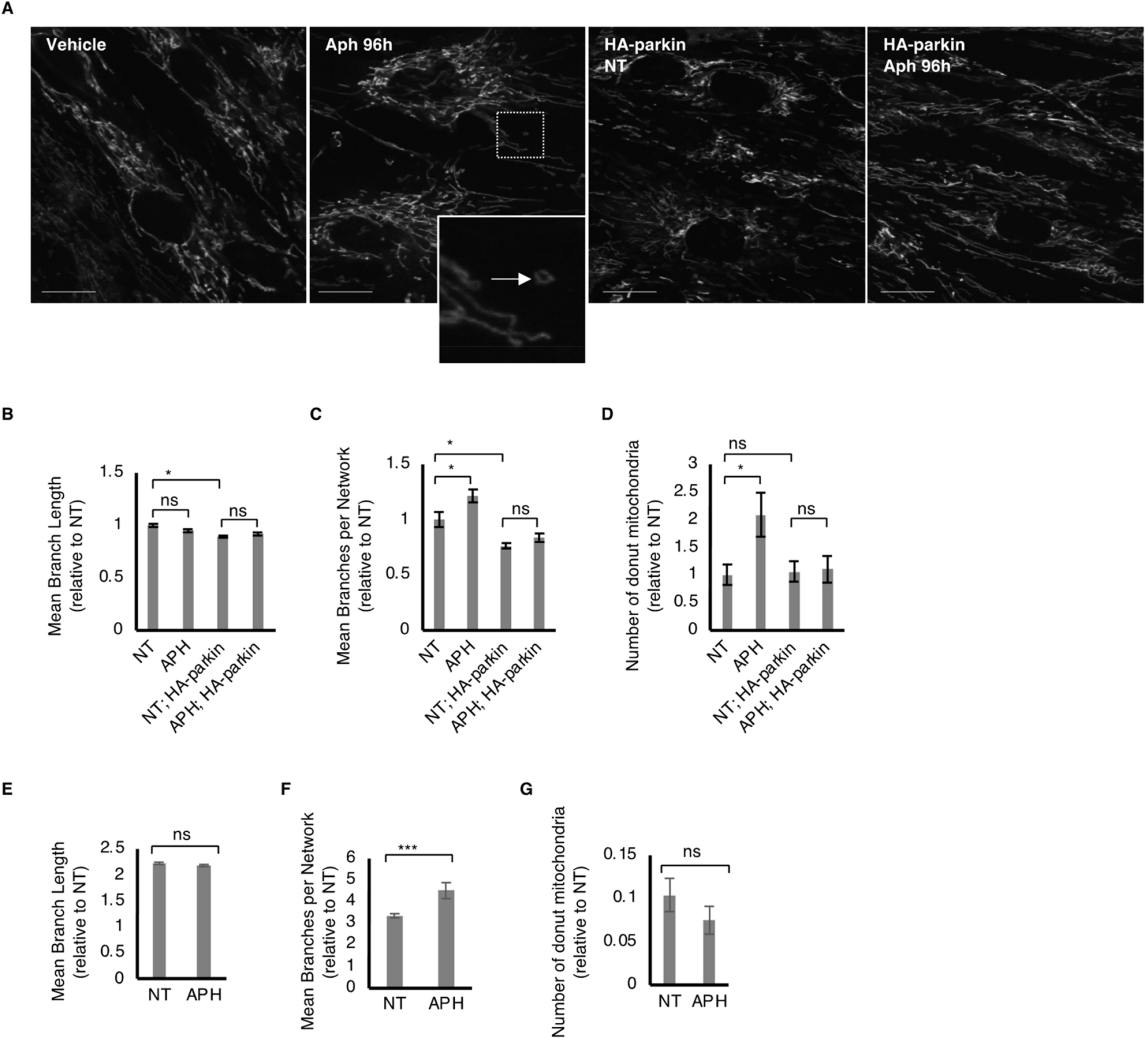
Constitutive replication stress and recovery causes morphological alteration in HDFs. (**A**) Representative images of morphological changes of mitochondria labeled with mitoTracker green. A arrow in the magnified image indicates a representation of donut mitochondria. Scale bar: 20 µm. (**B–D**) Summarized bar graphs of mean branch length (**B**), mean branches per network (**C**) or number of donut mitochondria relative to NT condition (**D**) in HDFs. Error bars indicates SEM of at least 60 cells for each condition were analyzed. ns: p > 0.05, *: p < 0.05 (one-way ANOVA). (**E–G**) Summarized bar graphs of mean branch length (**E**), mean branches per network (**F**) or number of donut mitochondria relative to NT condition (**G**) in HEK293A cells. Error bars indicates SEM of at least 60 cells for each condition were analyzed. ns: p > 0.05, ***: p < 0.005 (one-way ANOVA).

Next, we tested whether replication stress-exposed HDF showed changes in mitochondrial morphology. We found that both HDFs (Fig. 4A–D) and HEK293A cells (Fig. 4E–G) exposed to replication stress showed no significant changes in the mitochondrial tubular length, but increased the number of branches per network, indicating that the mitochondrial state in the cells exposed to replication stress becomes more complex rather than fragmented. Additionally, the number of donut-like mitochondrial structures, which may also refer to spheroid mitochondria, significantly increased under these conditions in HDFs but not in HEK293A cells (Fig. 4A, D and G). Donut mitochondria are stress-induced structures that may maintain mitochondrial quality not via autophagy, and Parkin is known to negatively regulate this pathway^30,31^. Overall, our data suggest that chronic replication stress causes a reduction in parkin protein, which does not primarily disrupt mitophagy activity, but causes mitochondrial dysfunction that may induce compensatory pathway(s) for mitochondrial homeostasis.

## Discussion

Although differentiated neurons are not exposed to replication stress due to a lack of proliferation, *PARK2* mutations, which comprise almost 15% of familial PD cases, are also responsible for sporadic PD^32^. Notably, it has been known that replication fork-slowing or stalling is prevalent in early embryogenesis^33^. As mild chronic replication stress causes a fixed reduction in parkin expression, our data supports the idea that de novo mutagenesis in *PARK2* locus during development may contribute to sporadic PD. In this study, we examined the effects of mild replication stress for 96 h for parkin expression. Considering the longer period of mammalian embryogenesis, our observations may recapitulate only a portion of the genomic alterations in stem or progenitor cells during development. Moreover, it may be more deleterious in the cells in precancerous lesions exposed to hypoxia, which induces replication stress and requires angiogenesis for further refilling of sufficient oxygen and building blocks. The process of hypoxic cells undergoing reperfusion and reoxygenation is also genotoxic as it causes DNA damage^34^. We expect that these replication stresses during proliferation under severe conditions and the reperfusion and reoxygenation processes may contribute to the complete loss of parkin expression in cancer cells which may promote further tumorigenesis.

In this study, we showed that chronic replication stress does not cause major reduction in CCCP-induced mitophagy. We speculate that residual parkin expression may be sufficient to carry out parkin-dependent mitophagy in APH-treated cells, but an alternative possibility is that parkin-independent mitophagy may also contribute to mitochondrial homeostasis under these conditions^35^. While parkin regulates mitophagy by ubiquitination of mitochondrial proteins via PINK1-dependent phosphorylation of ubiquitin^36^, parkin-independent mitophagy is mediated by mitophagy receptors NIX or FUNDC1^37^. These two possibilities are not mutually exclusive, so we speculate that both parkin-dependent and-independent pathways may cooperatively act in mitochondrial homeostasis in cells exposed to chronic replication stress. However, it should be noted that this does not necessarily mean that replication stress is negligible for mitophagy because of constitutive exposure of precancerous cells to replication stress caused by the expression of a wide variety of oncogenes, an early driver of genomic instability that is attributed to a plethora of factors^38^. We speculate that cells exposed to recurrent chronic replication stress cycles may experience loss of parkin expression, and that the parkin-independent pathway may become a major pathway for mitochondrial homeostasis.

In summary, we showed that exposure to chronic replication stress causes an irreversible change in endogenous parkin expression, causing mitochondrial dysfunction that is fully restored by ectopic parkin expression. Considering the pathophysiological role of mitochondrial dysfunction and the universality of replication stress^2,37^, we proposed parkin expression as a medication to alleviate any detrimental effects associated with exogenous or endogenous replication stress, such as chemotherapy or liver disease^39^.

## Materials and methods

### Antibodies and reagents

Antibodies against parkin (M230-3, MBL), NDUFA9 (ab14713, Abcam), DRP1 (8570, Cell Signaling Technologies), 8-oxoguanine (MOG-020P, JaICA), LC3 (PM036, MBL), TOM20 (Santa cruz biotechnologies, sc-17764) and phospho-Ser616-DRP1 (3455S, Cell Signaling Technologies) were used. Aphidicolin (01109811, FujiFilm), CCCP (034-16993, FujiFilm), Rotenone, MitoTracker Green (M7514, Invitrogen), and MitoSOX Red (M36008, Invitrogen) were used.

### Cell line, cell culture condition, and introduction of ectopic genes in HDF

Human dermal fibroblasts (HDFs) used in this study were purchased from Thermo Fisher Scientific (Human dermal fibroblasts, neonatal, Gibco, C0045C, Lot: 1186410), which were further immortalized by an hTERT expression construct (pBABE-neo-hTERT, Addgene plasmid #1774, a gift from Dr. Bob Weinberg)^40^. Briefly, HDFs were cultured in regular DMEM medium (043-30085, FujiFilm) supplemented with 10% FBS at 37 °C in a CO2 incubator. To introduce hTERT, PLAT-E cells were transfected with pBABE-neo-hTERT^40^ using Lipofectamine 3000 (L3000008, Invitrogene). At 48 h after transfection, supernatants were harvested and added to medium culturing HDFs seeded on a 6 cm dish. The HDFs were selected using neomycin (for hTERT). To introduce HA-parkin, hTERT expressing HDFs were transfected with pMXs-IP HA-Parkin (a gift from Dr. Noboru Mizushima, Addgene plasmid #38247)^41^. At 48 h after transfection, supernatants were harvested and added to medium culturing HDFs seeded on a 6 cm dish. The HDFs were selected using puromycin.

### Observation using confocal microscope and analysis of mitochondrial ROS

To measure mtROS levels, cells were labeled with MitoSOX Red (4 µM) and MitoTracker Green (100 nM) simultaneously for 15 min, washed, and transferred into a CO2 chamber using an FV10i confocal microscope (Olympus) with regular culture medium. For each experimental condition, four independent sites were captured every five minutes for 45 min. Images were analyzed using ImageJ software (Fiji, version 1.0, https://fiji.sc) with the JACoP plugin^42^ to measure the colocalization efficiency of the MitoSOX signal compared with the MitoTracker Green signal for each time point and obtain the averages of four independent sites for each experimental condition. At least three biologically independent experiments were performed and used for the statistical analysis.

To observe mitochondrial morphology, images of MitoTracker Green were captured using FV10i confocal microscope (Olympus) and analyzed using ImageJ with the MiNA plugin^43^.

### Western blotting

Cell lysate samples for western blotting were harvested using SDS sample buffer, run on SDS-PAGE gel, and transferred to PVDF membranes, followed by incubation with primary antibodies, washing with TBST, and incubation with appropriate secondary antibodies conjugated with the HRP enzyme. Chemiluminescent signals were obtained by chemical activation using ImmunoStar Zeta or ImmunoStar LD (FUJIFILM Wako Pure Chemical, 295-72404 and 290-69904, respectively), and detected using ImageQuant LAS-4000 (Cytiva).

### Immunofluorescent staining and microscopic observation

For immunostaining, HDFs or HEK293A cells were cultured on cover glasses in a 6 cm dish and fixed with 4% paraformaldehyde. After permeabilization with 50 µg/ml µg/mL digitonin in PBS for 10 min, the cells were blocked with PBS containing 0.2% gelatin for 20 min, incubated with primary antibodies, washed twice with PBS, and incubated with secondary antibodies with DAPI in PBS supplemented with 0.2% gelatin for 60 min. The coverslips were mounted with ProLong Gold antifade reagent (P36934, Invitrogen) and observed under a fluorescence microscope (IX71 and DP80, Olympus). The obtained images were analyzed to count the number of autophagosomes or their co-localization using ImageJ software (Fiji, version 1.0, https://fiji.sc) with the JACoP plugin^42^. To visualize mitochondria, cells were cultured supplemented with 50 nM MitoTracker Red (M7512, Thermo Fisher) for 30 min before harvest. To visualize 8-oxoguanine, fixed cells were incubated with an antibody against 8-oxoguanine (mouse monoclonal, MOG-020P, JaICA), followed by the corresponding secondary antibody staining.

### RNA-seq analysis

HDF cells were exposed to 300 nM APH for 96 h, followed by 96 h of recovery culture without APH and harvest for RNA extraction. Total RNA was obtained using the PureLink RNA Mini Kit (12183018A, Invitrogen) according to the manufacturer's protocol. After library generation, large scale sequencing was performed using the illumine NovaSeq6000. Reference genome and gene model annotation files were downloaded directly from the genome website browser (NCBI/UCSC/Ensembl). An index of the reference genome was constructed using STAR, and paired-end clean reads were aligned to the reference genome using STAR (v2.5). STAR used the Maximal Mappable Prefix (MMP) method, which can generate accurate mapping results for junction reads. HTSeq v0.6.1 was used to count the number of mapped reads for each gene. Differentially expressed genes (DEGs) were detected with the thresholds of |log2FC (Fold Change)|> 1 and adjusted p value < 0.05 by Benjamini and Hochberg (BH) method.

### Statistical analysis

Quantitative data represent the mean ± standard error from at least three biological independent experiments. Statistical analyses were performed using ordinary one-way ANOVA analysis or Student’s t-test.

### Supplementary Information

Supplementary Table S1.

Supplementary Figures.

## Data Availability

All the raw data from experiments in this manuscript are available on request for T.K. (t-kawabata@nagasaki-u.ac.jp).
