# Supplementary material for: Chronic replication stress invokes mitochondria dysfunction via impaired parkin activity
Source: Sci Rep. 2024 Apr 3;14:7877. doi: 10.1038/s41598-024-58656-w (PMC10991263; doi:10.1038/s41598-024-58656-w)

Supplementary data

Chronic replication stress invokes mitochondria dysfunction via impaired parkin activity

Tsuyoshi Kawabata [1*], Reiko Sekiya[1], Shinji Goto[1], Tao-Sheng Li[1]

1 Department of Stem Cell Biology, Atomic Bomb Disease Institute, Nagasaki University, Nagasaki, Japan.

*: Corresponding author: t-kawabata@nagasaki-u.ac.jp

A

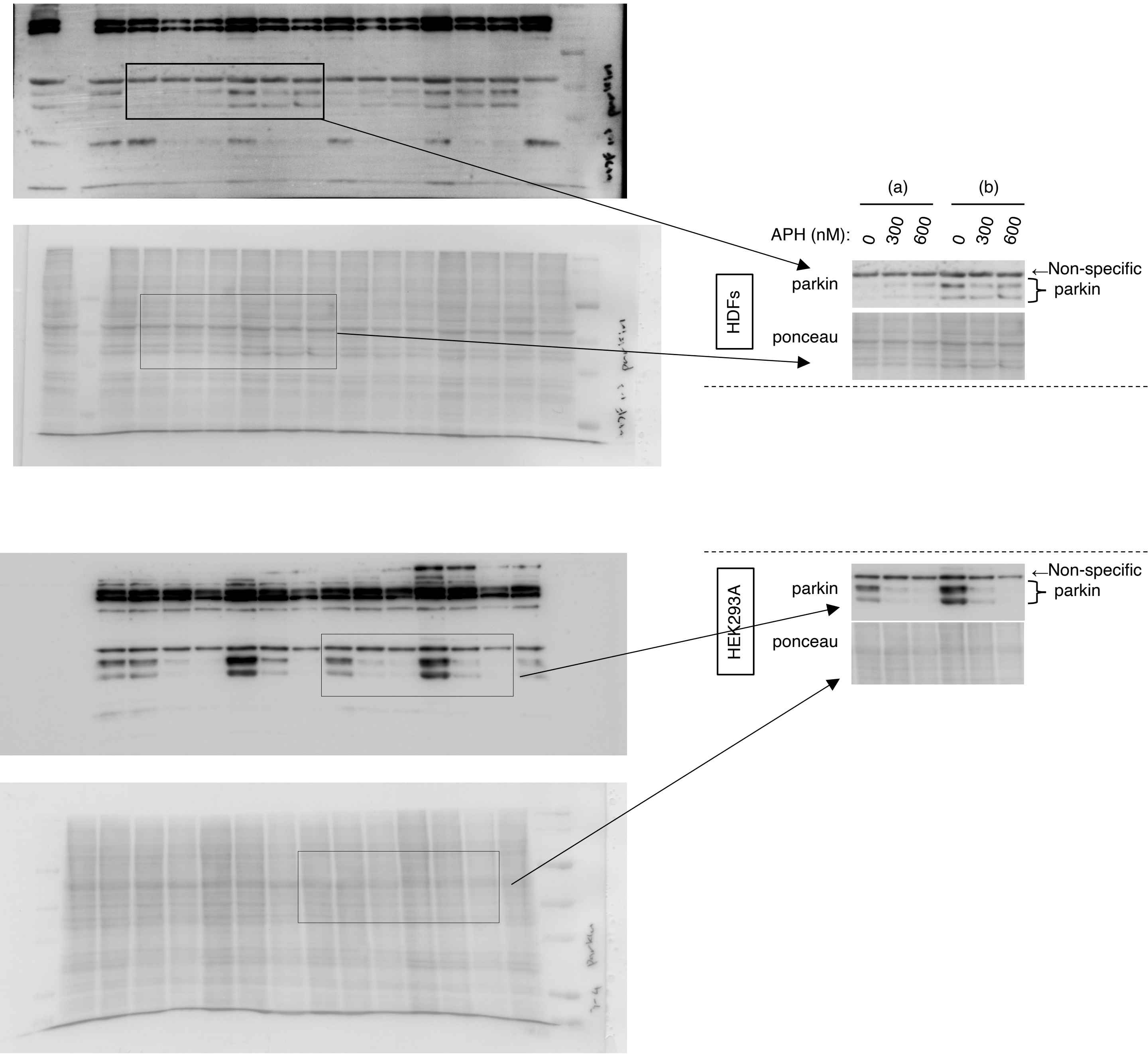
(a)
(b)
APH (nM):
0
300
600
0
300
600
HDFs
parkin
←Non-specific
parkin
ponceau
HEK293A
parkin
←Non-specific
parkin
ponceau

B

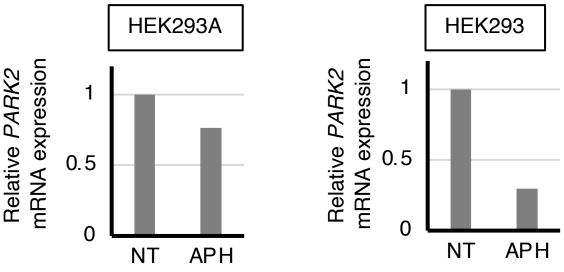
HEK293A
Relative PARK2 mRNA expression
1
0.5
0
NT
APH
HEK293
Relative PARK2 mRNA expression
1
0.5
0
NT
APH

C

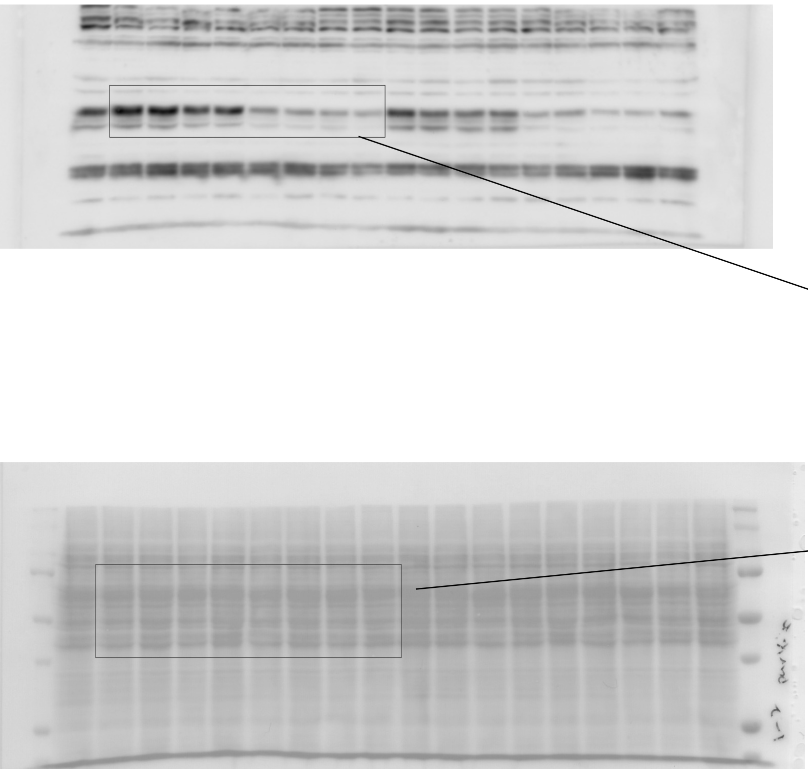

D

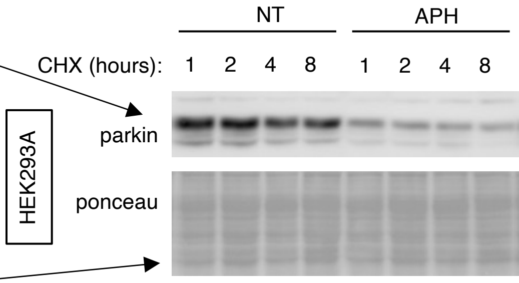

Fig. S1 Full length images of an immunoblot of parkin and ponceau S-stained membrane.

A) Black boxes indicate the cropped images of the immunoblot and the membrane shown in Figure 1B.

B) Relative expression of mRNA of *PARK2* gene in HEK293A (left) and HEK293 (right) cells treated with chronic 300nM APH, obtained from previously published data of RNA-seq (left) and microarray (right), respectively.

C) Black boxes indicate the cropped images of the immunoblot and the membrane shown in Figure 1D.

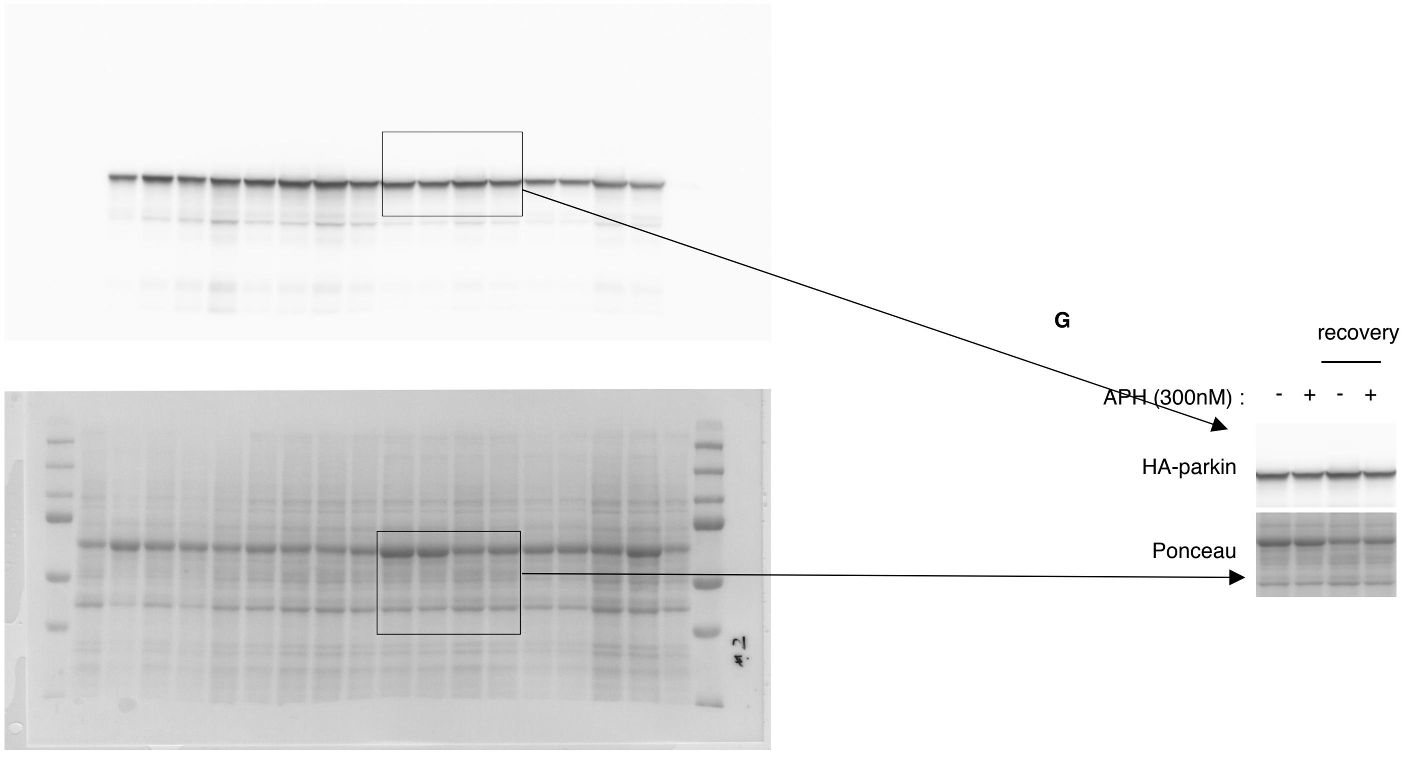

Fig. S2 Full length images of an immunoblot of parkin and ponceau S-stained membrane. Black boxes indicate the cropped images of the immunoblot and the membrane shown in Figure 2G.

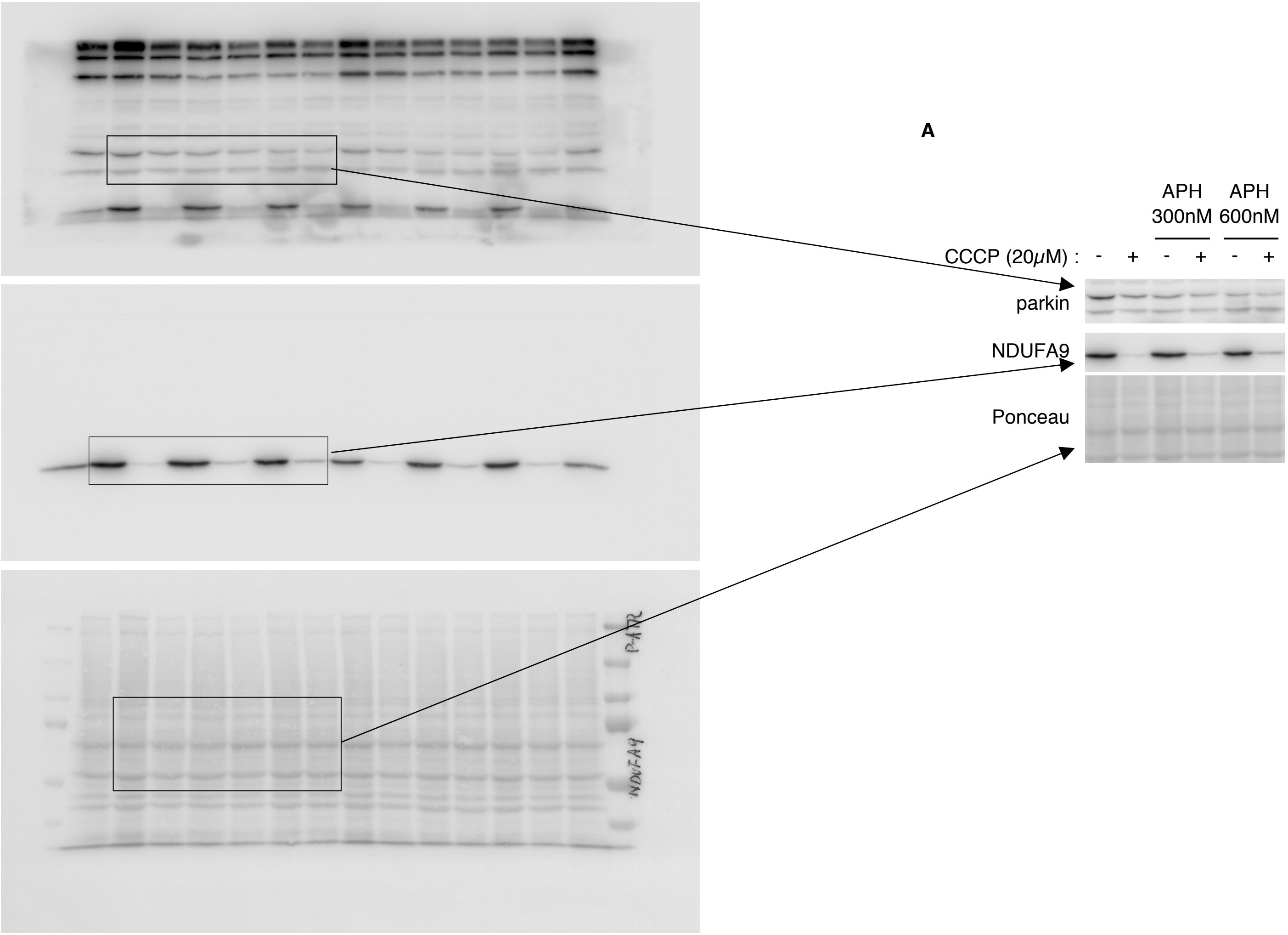

Fig. S3 Full length images of immunoblots of parkin and NDUFA9 and ponceau S-stained membrane. Black boxes indicate the cropped images of the immunoblot and the membrane shown in Figure 3A. Note that the blot of parkin is the same as the blot of NDUF9A, whose antibodies are stripped and reused for parkin immunoblot.

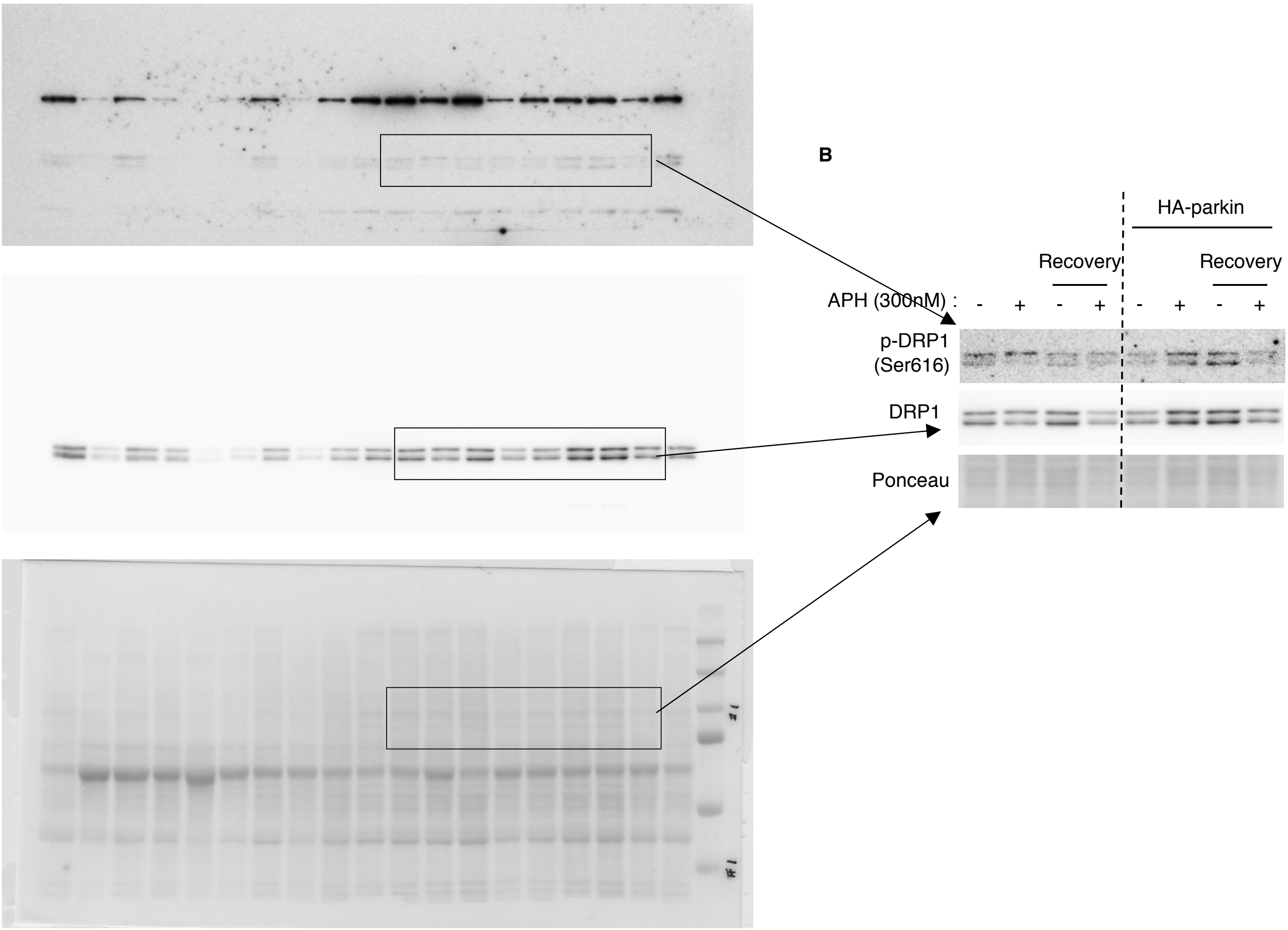

Fig. S4 Full length images of immunoblots of NDUFA9 and phospho-NDUFA9 and ponceau S-stained membrane. Black boxes indicate the cropped images of the immunoblot and the membrane shown in Figure 3B. Note that the blot of DRP1 is the same as the blot of p-DRP1, whose antibodies are stripped and reused for DRP1 immunoblot.

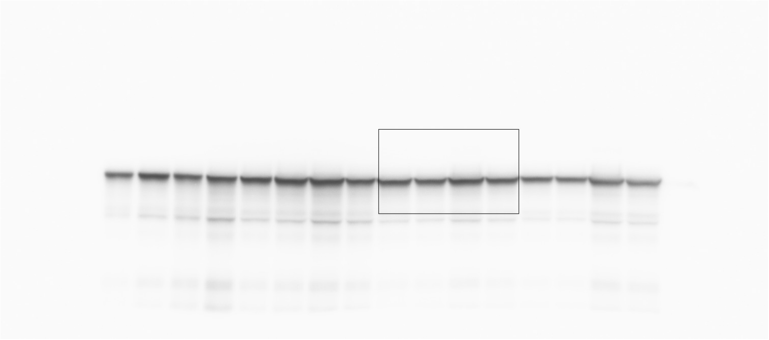

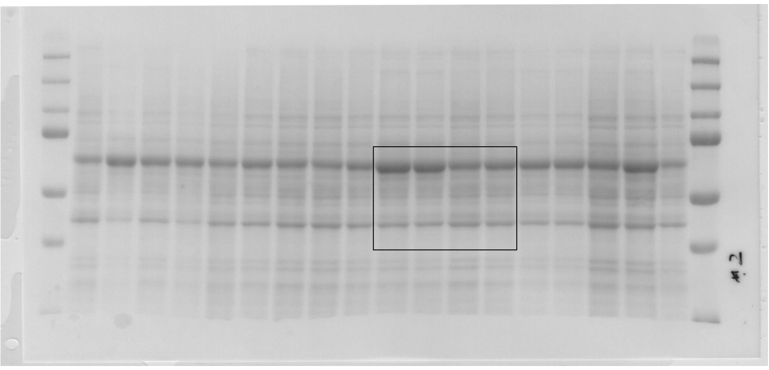

Fig.1B parkin

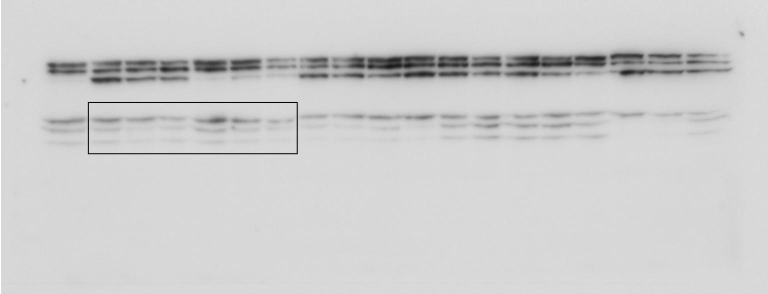

Fig.1B ponceau

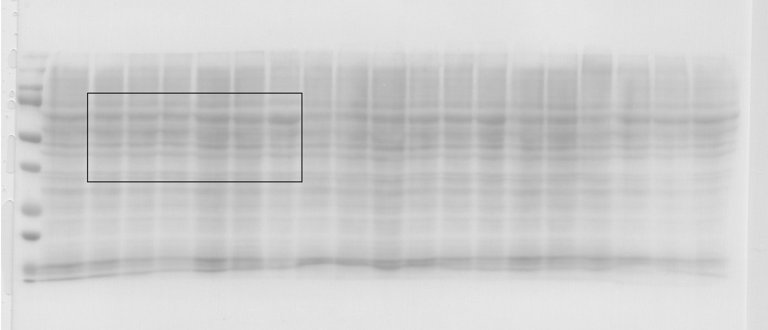

Fig.2G parkin

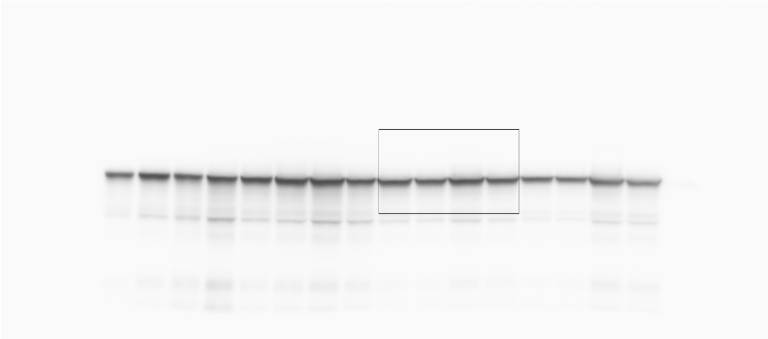

Fig.2G ponceau

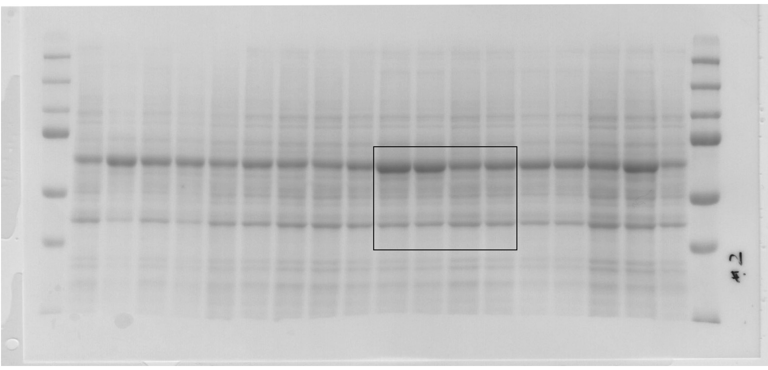

B

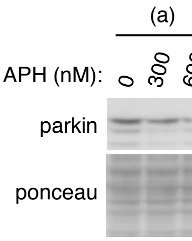

Fig.3A NDUFA9

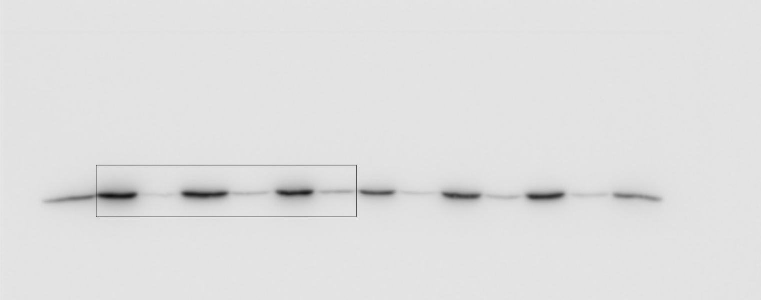

Fig.3A parkin
*same blot as NDUFA9.
The blot of NDUFA9 was stripped and used for anti-parkin blot.

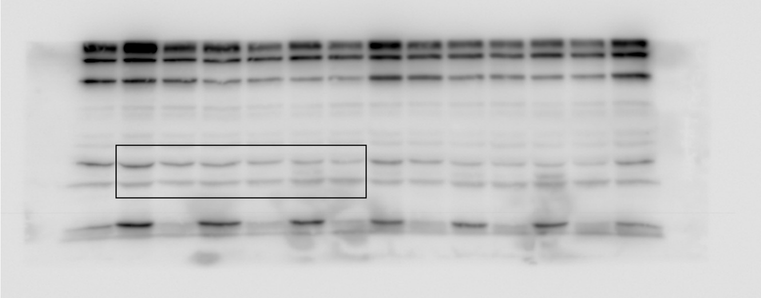

Fig.3B ponceau

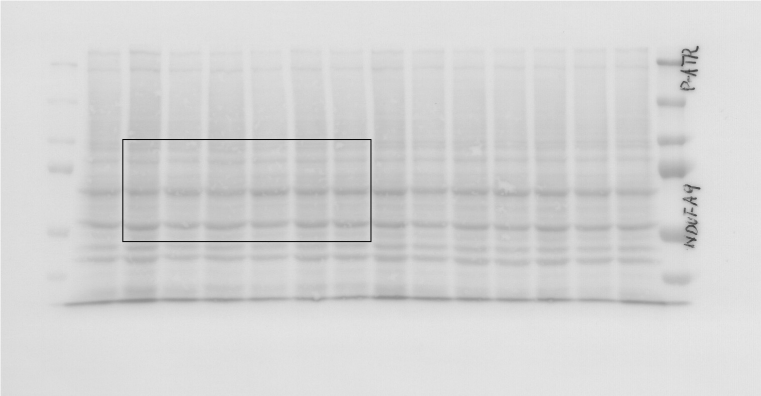

Fig.3B p-DRP1

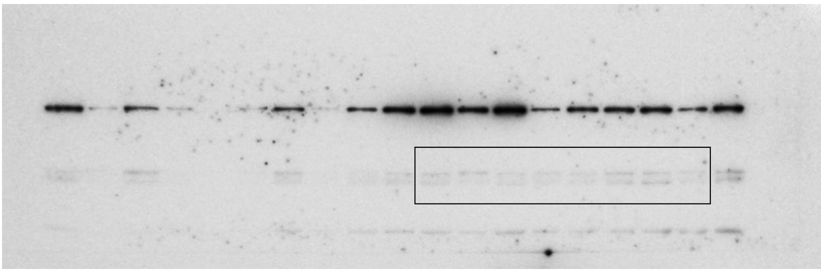

Fig.3B DRP1
*same blot as p-DRP1.
The blot of p-DRP1 was stripped and used for anti-DRP1 blot.

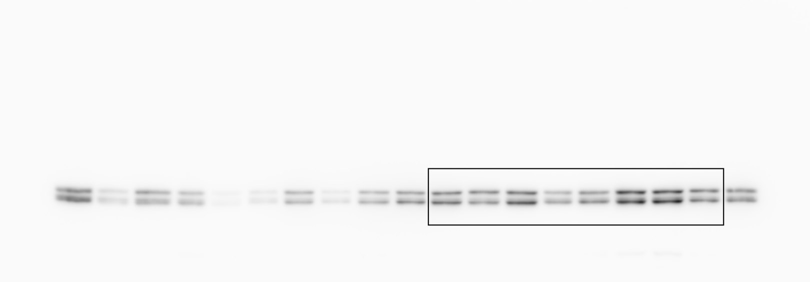

Fig.3B ponceau

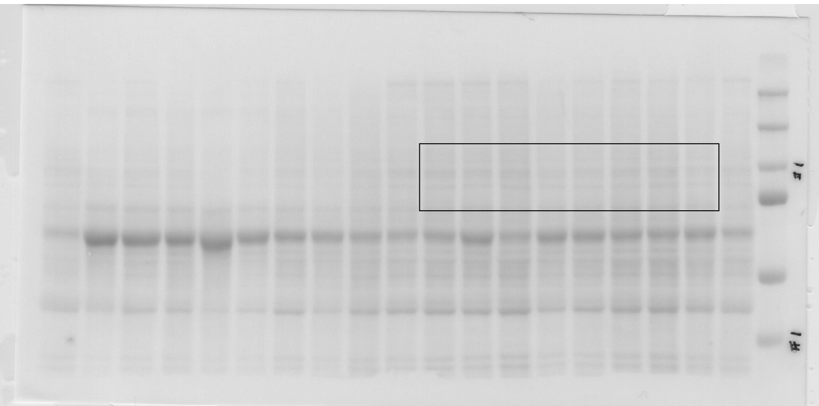

Supplement: Supplementary file 2 — Supplementary Figures. [file 41598_2024_58656_MOESM2_ESM.pdf]
